# Mortality Not Increased in Patients With Nonfunctional Adrenal Adenomas: A Matched Cohort Study

**DOI:** 10.1210/clinem/dgad074

**Published:** 2023-02-17

**Authors:** Albin Kjellbom, Ola Lindgren, Malin Danielsson, Henrik Olsen, Magnus Löndahl

**Affiliations:** Department of Endocrinology, Skåne University Hospital, Lasarettsgatan 15, SE-221 85, Lund, Sweden; Faculty of Medicine, Department of Clinical Sciences Lund, Lund University, Office DCSL, Hämtställe 66, BMC F12, SE-221 84, Lund, Sweden; Department of Endocrinology, Skåne University Hospital, Lasarettsgatan 15, SE-221 85, Lund, Sweden; Faculty of Medicine, Department of Clinical Sciences Lund, Lund University, Office DCSL, Hämtställe 66, BMC F12, SE-221 84, Lund, Sweden; Department of Endocrinology, Skåne University Hospital, Lasarettsgatan 15, SE-221 85, Lund, Sweden; Faculty of Medicine, Department of Clinical Sciences Lund, Lund University, Office DCSL, Hämtställe 66, BMC F12, SE-221 84, Lund, Sweden; Department of Endocrinology, Ängelholm Hospital, Ängelholms sjukhus, SE-262 81, Ängelholm, Sweden; Department of Endocrinology, Skåne University Hospital, Lasarettsgatan 15, SE-221 85, Lund, Sweden; Faculty of Medicine, Department of Clinical Sciences Lund, Lund University, Office DCSL, Hämtställe 66, BMC F12, SE-221 84, Lund, Sweden

**Keywords:** adrenal incidentaloma, adrenocortical adenoma, humans, mortality, cortisol

## Abstract

**Context:**

Mild autonomous cortisol secretion (MACS) is associated with increased mortality in patients with adrenal incidentalomas, but little is known regarding the potential risk associated with nonfunctional adrenal adenomas (NFAA), which constitute the majority of adrenal incidentalomas.

**Objective:**

Compare mortality risk in patients with NFAA, and different levels of MACS, to matched controls.

**Method:**

This was a retrospective matched cohort study. All patients referred to 2 endocrine centers in southern Sweden because of an adrenal incidentaloma between 2005 and 2015 were enrolled. Controls (3:1) matched for sex, age, and residency were included. Primary endpoint was all-cause mortality. Outcome data were obtained from the Cause of Death Register. Patients were grouped according to cortisol level post 1-mg dexamethasone suppression test (cortisol_DST_) (<50 (NFAA), 50-82, 83-137, and ≥138 nmol/L).

**Results:**

1154 patients and 3462 matched controls were included. During a median follow-up of 6.6 years, 210 patients and 505 controls died. There were no statistically significant differences in mortality between patients with NFAA and their controls (HR 1.13 [0.87-1.46]) whereas mortality was increased compared to controls in patients with cortisol_DST_ 83-137 (HR 1.99 [1.38-2.88]) and ≥138 nmol/L (HR 4.09 [2.41-6.93]). Likewise, the mortality risk was increased in patients younger than 65 years with cortisol_DST_ 50-82 nmol/L compared with controls (HR 2.33 [1.30-4.17]).

**Conclusion:**

NFAA does not seem to pose a clinically relevant risk for increased mortality in patients with adrenal incidentalomas while patients with MACS, and especially younger patients and those with cortisol_DST_ ≥83 nmol/L, have significantly increased mortality risk compared with matched controls.

With the widespread use of imaging studies, adrenal incidentalomas are a common clinical issue, affecting up to 5% of the adult population (1, 2). The majority of adrenal incidentalomas are nonfunctional adenomas (NFAA), but many cause mild autonomous cortisol secretion (MACS), also known as autonomous cortisol secretion. The MACS diagnosis is based on the results of the 1-mg dexamethasone suppression test in patients with an adrenal adenoma. A posttest cortisol <50 nmol/L excludes MACS and, in the absence of signs of other adrenal hormone excess, defines NFAA, while a confirmed cortisol ≥50 nmol/L suggests MACS (1). Several studies have shown an association between MACS and increased mortality, not least in people younger than 65 years of age (3-7). There is also some indirect evidence suggesting that NFAA might be associated with an increase in cardiometabolic risk (8). Since previous studies investigating mortality risk in patients with MACS used NFAA as reference group, a potential risk associated with NFAA might have been overlooked (3-7).

Imaging studies are an integral part of modern health care, providing great benefits for patients. It also entails that patients with adrenal incidentalomas will continue to be common, probably increasingly so, in clinical medicine. Since NFAA is the most frequent finding in patients with adrenal incidentalomas, evaluating mortality risk in these patients is of clinical importance.

To our knowledge, there are no published data on mortality in patients with NFAA or MACS compared with matched controls. Our objective was to compare mortality risk in patients with NFAA, and different levels of MACS, relative to matched controls.

## Methods

We did a population-based matched cohort study evaluating mortality risk in patients with adrenal incidentalomas compared with matched controls. The endocrine referral centers at Helsingborg Regional Hospital and Skåne University Hospital, both located in southern Sweden, constituted the study sites. Our study population consisted of inhabitants in the catchment areas of these hospitals. During the study period, regional guidelines recommended that all patients with an adrenal incidentaloma be referred to the endocrine center at their regional hospital. All adult patients (≥18 years of age) examined for previously unknown adrenal adenomas found as adrenal incidentalomas between January 1, 2005, and September 15, 2015, were enrolled.

Patients underwent 1-mg dexamethasone suppression test, with fasting plasma cortisol being measured at 8 Am. Pheochromocytoma, primary aldosteronism, and cancer were evaluated clinically, biochemically, and by radiology according to current guidelines. Exclusion criteria were adrenal incidentalomas <10 mm, metastatic cancer, treatment with oral glucocorticoids, systemic estrogen treatment, medication affecting dexamethasone metabolism, nonadenoma lesion such as myelolipoma or hemorrhage, pheochromocytoma, primary aldosteronism, 1-mg dexamethasone suppression test not performed, and clinical Cushing syndrome.

The authors collected clinical and biometrical data from the electronic medical records (A.K., H.O., and O.L.). Before outcome data was obtained, the database was locked.

Patients were grouped according to predefined levels of cortisol after 1-mg dexamethasone suppression test (cortisol_DST_) (<50 [NFAA], 50-82, 83-137, and ≥138 nmol/L). To convert cortisol to µg/dL divide values by 27.5862.

Controls were randomly selected from the general population in a 3:1 ratio, using the National Population Registry provided by Statistics Sweden. Each included patient was assigned 3 individual controls matched for sex, age, and residency. Controls did not undergo 1-mg dexamethasone suppression test, and other baseline information than the matching factors were not available in controls.

A 1-step competitive immunoassay (Cobas, Roche Diagnostics) was used to analyze plasma cortisol. The reference range was 171 to 536 nmol/L, the coefficient of variation 2.1% at 94.9 nmol/L, and the detection limit was 0.5 nmol/L.

The primary outcome was all-cause mortality, and the secondary outcome was cause-specific mortality. Outcome data were obtained from the National Board of Health and Welfare Cause of Death Register. Cause of death in the register was coded according to the International Classification of Diseases, Tenth revision (ICD-10). Cause-specific mortality was analyzed in 4 categories based on ICD-10 codes, cardiovascular (ICD-10 codes, I2-9 excluding I38), cancer (ICD-codes, C0-9), infections (ICD-10 codes, J1, I38, A419), and other causes (remaining ICD-10 codes).

Patients were followed from the date of 1-mg dexamethasone suppression test until adrenalectomy, emigration, death, or December 31, 2018. Controls were followed until emigration, death, or December 31, 2018.

Kaplan-Meier plots, with log rank test, were used to visualize unadjusted all-cause mortality. Cox regression, including the covariates sex and age, was used to calculate hazard ratios (HR) and 95% CI between patients and controls, grouped according to cortisol_DST_. Interaction analysis was performed by expanding the model with a 3-way full factorial term for each cortisol_DST_ group. Secondary endpoints were only analyzed if there was a statistically significant difference regarding the primary endpoint within the respective groups. The Fine and Gray method was used to account for competing risks when analyzing the secondary endpoints (9). Differences in baseline characteristics between patient groups were analyzed: categorical data were analyzed using the Chi-square test, and continuous data using the Mann-Whitney test. A two-tailed *P* value <.05 was considered statistically significant. Proportional hazard assumption was tested with Schoenfeld residuals. Descriptive data were given as median and interquartile range (IQR) or percentages. Statistical analysis was performed using StataCorp. 2021. *Stata Statistical Software: Release 17*. College Station, TX: StataCorp LLC.

The study was approved by the Regional Ethical Review Board in Lund, Sweden, and registered at clinicaltrials.gov (NCT03919734).

The funding sources had no influence on the design, data analysis, interpretation of the results, or the decision to submit the manuscript for publication.

## Results

**Figure 1.**
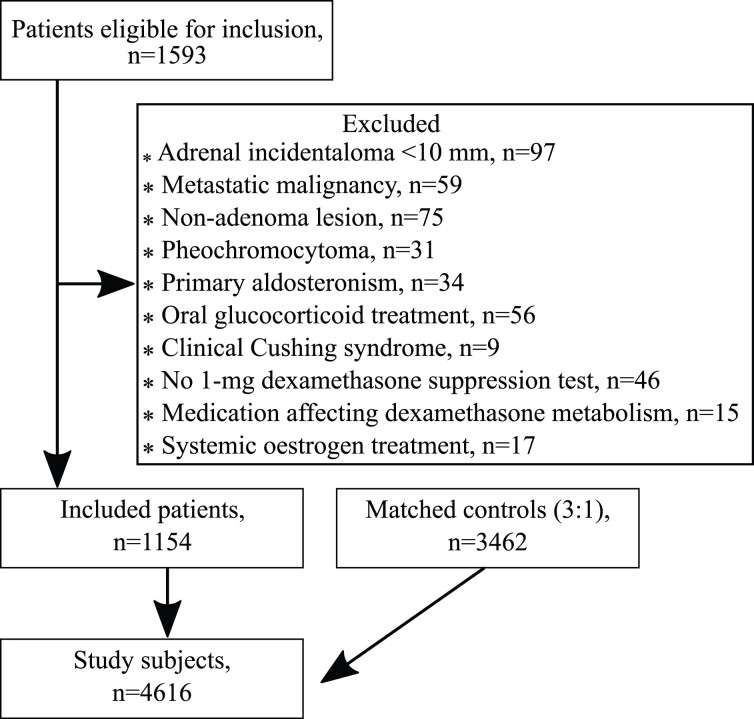
Inclusion flow diagram.

1154 patients aged 65.1 years (57.5-71.4) (59.5% women) and 3462 matched controls (age 65.1 (57.6-71.4); 59.5% women) were included. Among the patients, 632 (54.8%) had a NFAA (Fig. 1). Patients with cortisol_DST_ ≥50 nmol/L were significantly older, had lower body mass index, higher prevalence of hypertension, and previous cardiovascular disease, and they were more often smokers than patients with NFAA, while there were no significant differences in the prevalence of diabetes mellitus, or sex. Differences in baseline characteristics between the 3 patient groups with cortisol_DST_ ≥50 nmol/L did not reach statistical significance. Controls were well-matched; an overview of patient and control characteristics are shown in Table 1. Between-group analysis of the patient groups has previously been published (6).

**Table 1.** Group characteristics

	NFAA (Cortisol_DST_ < 50 nmol/L)	Cortisol_DST_ 50-82 nmol/L	Cortisol_DST_ 83-137 nmol/L	Cortisol_DST_ ≥ 138 nmol/L
**Patients, n**	**632**	**305**	**134**	**83**
Age, years	63.0 (54.8-69.6)	66.0 (60.7-73.8)*^c^*	67.9 (60.9-75.1)*^c^*	68.9 (61.3-74.6)*^c^*
Women, % (n)	57.1% (361)	63.6% (194)	65.7% (88)	53.0% (44)
BMI, kg/m^2*^d^*^	28.2 (25.2-32.3)	26.9 (23.5-30.9)*^c^*	26.8 (23.7-30.6)*^b^*	25.6 (21.4-29.6)*^c^*
Smoker, % (n)	30.4% (192)	40.0% (122)*^b^*	50.0% (67)*^c^*	48.2% (40)*^b^*
Hypertension, % (n)	45.4% (287)	59.0% (180)*^c^*	65.7% (88)*^c^*	68.7% (57)*^c^*
Diabetes mellitus, % (n)	16.3% (103)	20.7% (63)	22.4% (30)	16.9% (14)
Cardiovascular disease, % (n)	17.4% (110)	24.6% (75)*^a^*	25.4% (34)*^a^*	26.5% (22)*^a^*
All-cause mortality, n/1000 person-years, (%, n)	18.0 (12.3%, n = 78)	31.6 (20.7%, n = 63)	54.1 (32.8%, n = 44)	68.8 (30.1%, n = 25)
Cause-specific mortality, % (n)				
• Cardiovascular disease	4.6% (29)	7.2% (22)	13.4% (18)	13.3% (11)
• Cancer	3.2% (20)	6.2% (19)	9.7% (13)	6% (5)
• Infection	0.5% (3)	1% (3)	1.5% (2)	1.2% (1)
Other	4.1% (26)	6.2% (19)	8.2% (11)	9.6% (8)
Follow-up, years	6.6 (4.9-8.8)	6.4 (4.1-8.9)	6.2 (3.5-8.3)	4.2 (1.8-6.6)
**Controls, n**	**1896**	**915**	**402**	**249**
Age, years	63.1 (54.7-69.6)	66.0 (60.6-73.9)	67.9 (60.9-75.0)	68.6 (61.6-74.4)
Women, % (n)	57.1% (1083)	63.6% (582)	65.7% (264)	53.0% (132)
All-cause mortality, n/1000 person-years, (%, n)	16.0 (11.2%, n = 213)	27.2 (18.7%, n = 171)	29.5 (20.6%, n = 83)	22.8 (15.3%, n = 38)
Cause-specific mortality, % (n)				
• Cardiovascular disease	2.7% (51)	6.2% (57)	7.5% (30)	4.8% (12)
• Cancer	4.1% (77)	6.3% (58)	6.0% (24)	2.8% (7)
• Infection	0.6% (11)	0.9% (8)	1.5% (6)	1.2% (3)
• Other	3.9% (74)	5.2% (48)	5.7% (23)	6.4% (16)
Follow-up, years	6.6 (4.8-9.1)	6.8 (4.3-9.0)	6.9 (4.8-9.3)	6.1 (4.3-8.8)

Patients grouped according to predefined cortisol_DST_ levels, controls individually matched to each patient by sex, age, and residency (3:1). Continuous data are given as median and interquartile range (IQR), binary data as % and number (n). Statistical differences in baseline characteristics were calculated between patient groups.

Abbreviations: BMI, body mass index; NFAA, nonfunctional adrenal adenoma.

*P* < 0.05 compared to NFAA.

*P* < .01 compared to NFAA.

*P* < .001 compared to NFAA. Conversion factor: To convert cortisol to µg/dL divide values by 27.5862.

Available in 613, 301, 133, and 82 patients.

### All-cause Mortality

**Figure 2.**
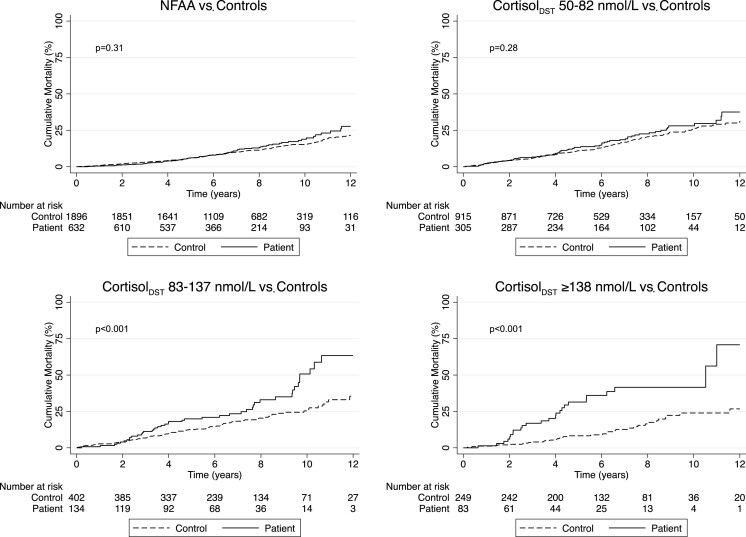
Unadjusted all-cause mortality. Kaplan-Meier plots of unadjusted cumulative mortality for patients and controls divided by cortisol_DST_ groups. *P* value derived from log rank test. Plots are truncated at 12 years.

During a median follow-up period of 6.3 years (4.3-8.7) in patients and 6.7 years (4.7-9.1) in controls, 210 (18.2%) patients and 505 (14.6%) controls died (Figure 2). There were no statistically significant differences in mortality between patients with NFAA or cortisol_DST_ 50-82 nmol/L and their controls, HR 1.13 (95% CI, 0.87-1.46) and 1.13 (0.85-1.51), respectively. HR for patients with cortisol_DST_ <83 nmol/L (NFAA and cortisol_DST_ 50-82 combined) compared with controls was 1.13 (0.93-1.37).

In patients with cortisol_DST_ 83-137 or ≥138 nmol/L, mortality was increased compared to controls, HR 1.99 (1.38-2.88) and HR 4.09 (2.41-6.93). Analyzed as a single group, cortisol_DST_ ≥ 83 nmol/L, HR was 2.48 (1.84-3.35) for all-cause mortality (Table 2). There was no violation of the proportional hazard assumption.

**Table 2.** Hazard ratios for all-cause and cause-specific mortality

	All-cause mortality HR (95% CI)	Cardiovascular mortality HR (95% CI)	Cancer mortality HR (95% CI)	Infection mortality HR (95% CI)	Other mortality HR (95% CI)
NFAA (Cortisol_DST_ < 50 nmol/L)	1.13 (0.87-1.46)				
Cortisol_DST_ 50-82 nmol/L	1.13 (0.85-1.51)				
Cortisol_DST_ 83-137 nmol/L	1.99 (1.38-2.88)	1.96 (1.10-3.49)	1.83 (0.93-3.61)	1.03 (0.21-5.09)	1.59 (0.78-3.27)
Cortisol_DST_ ≥ 138 nmol/L	4.09 (2.41-6.93)	4.28 (1.81-10.15)	2.83 (0.88-9.09)	1.26 (0.12-13.53)	2.02 (0.87-4.68)
Cortisol_DST_ < 83 nmol/L	1.13 (0.93-1.37)				
Cortisol_DST_ ≥ 83 nmol/L	2.48 (1.84-3.35)	2.51 (1.56-4.04)	2.01 (1.12-3.62)	1.08 (0.29-4.06)	1.73 (1.00-2.99)

Hazard ratios (HR) and 95% CI for patients compared to controls. HR adjusted for sex and age. HR for cause-specific mortality calculated using the Fine and Gray method for competing risks.

### Cause-specific Mortality

The risk of cardiovascular mortality was significantly increased in patients with cortisol_DST_ 83-137 and ≥138 nmol/L compared with their controls, HR 1.96 (1.10-3.49) and 4.28 (1.81-10.15). In patients with cortisol_DST_ ≥ 83 nmol/L, cancer associated mortality was significantly increased compared to controls, HR 2.01 (1.12-3.62) (Table 2). Overall, 57 patients and 166 controls died because of cancer. The most prevalent cancers were lung and colon cancer. In patients, lung cancer constituted 33% (n = 19), and colon cancer 12% (n = 7) of deaths due to cancer. The corresponding percentages in controls were 23% (n = 38) and 13% (n = 22). In patients with cortisol_DST_ ≥ 83 nmol/L, 5 and 3 patients died due to lung and colon cancer, respectively; the corresponding numbers in their controls were 5 and 2, respectively.

### Subgroup Analysis

Interaction analysis showed a significant interaction between age and patients, in the cortisol_DST_ 50-82 nmol/L group (*P* = .049). Patients and their respective controls were divided in 2 groups according to age: younger than 65, or 65 years of age and older (7, 10). In a subgroup analysis, a significant increase in mortality risk compared with controls was seen in patients younger than 65 years of age (HR 2.33 [1.30-4.17]) but not in those 65 or older (0.92 [0.65-1.28]) (Table 3).

**Table 3.** Subgroup analysis

Cortisol_DST_ 50-82 nmol/L	Age, years	N	Events (%)	All-cause mortality HR (95% CI)
Patient	<65	131	19 (14.5)	2.33 (1.30-4.17)
Control		393	28 (7.1)	
Patient	≥65	174	44 (25.3)	0.92 (0.65-1.28)
Control		522	143 (27.4)	

Hazard ratios (HR) and 95% CI for patients compared to controls. HR adjusted for sex and age.

## Discussion

This is the first study to compare mortality risk in patients with NFAA and MACS with matched controls. The results indicate that there is no association between NFAA and increased mortality risk.

MACS is a continuous state, and it is possible that patients with adrenal incidentalomas and cortisol_DST_ <50 nmol/L have subtle cortisol hypersecretion rendering the many observed metabolic alterations in this patient group (11-17). Results from this study indicate that these alterations do not translate into a clinically relevant increase in mortality, at least not in a 10-year perspective. Thus, these findings support current guidelines recommending no follow-up for patients with NFAA (1). The results regarding patients with cortisol_DST_ ≥50 nmol/L add to previous evidence of an association between MACS and mortality, with a positive correlation between cortisol_DST_ and increase in risk, where a cortisol_DST_ level ≥83 nmol/L seems to indicate a clinically relevant increase in risk (3-7).

Analysis of cause-specific mortality showed a higher mortality due to cardiovascular disease and cancer in patients with cortisol_DST_ level ≥83 nmol/L compared with controls. The link between cortisol excess and cardiovascular disease is well established, although not fully understood (18). The association between MACS and cancer has been observed in one previous study, while theoretical links are plausible, this association needs to be studied further (5).

In this study we found evidence suggesting age disparity regarding the risk of discrete MACS (cortisol_DST_ 50-82 nmol/L). The results indicate that the effects of discrete MACS afflict younger patients to a greater extent. Hypothetical explanations could be a more severe phenotype of MACS in younger patients or cortisol being a more robust predictor in the absence of manifest comorbidities. The latter was proposed in one previous study indicating age-dependent risk of MACS. The study, by Deutschbein et al, showed that women under the age of 65 years had the highest mortality risk associated with MACS (7). Our results to some extent corroborate these findings, MACS being a stronger risk-factor in patients <65 years of age; however, in contrast, we found no significant interaction for sex in our study.

Sweden has a universal healthcare system, and the study sites covered all inhabitants in the catchment areas; thus, with enrollment of all referred patients during the study period, the risk of selection bias was reduced. We believe the study size and the before mentioned factors render strong external validity. The observed lack of statistically significant difference in all-cause mortality between patients with NFAA, or cortisol_DST_ 50-82, and their controls does not exclude a difference, but given the sample size, we deem the study to have sufficient power to detect a clinically relevant difference regarding the primary endpoint (19-21). The access to reliable registers makes outcome data on the primary endpoint credible, as the Cause of Death Register is more than 99% complete (22). It may be a fair assumption that the prevalence of adrenal incidentalomas and MACS, as with many other diseases, is associated with socioeconomic factors (23, 24). In 2014-2018 the between-municipality variation in life expectancy in our catchment areas was 5 years (25). By matching controls by residency, we reduced the potential influence of socioeconomic factors.

We acknowledge that the study has limitations. While Sweden has a universal health care system with guidelines recommending all patients with adrenal incidentalomas to be referred to an endocrine center, the most fragile patients with a short life expectancy might have yet to be referred, exemplifying possible referral bias. The study design, excluding patients with (for example) metastatic malignancy and oral glucocorticoid treatment, might also have led to a healthy entrant effect (26). The prevalence of classical cardiovascular risk factors, such as smoking and diabetes mellitus, were higher in patients, both in the NFAA and MACS group, than in the general Swedish population, thus constituting possible confounders (27, 28). These confounders would lead to an overestimation of the risk associated with NFAA and MACS. As the numbers are small, and the events few, this should especially be taken into consideration when interpreting the results of the subgroup analysis. Further, subgroup analyses often suffer from being underpowered, urging inference to be made with caution. One should also take into account that the cause-specific mortality reported to the Cause of Death Register might be inaccurate in up to 15% of cases (22, 29). Post hoc analysis, excluding patients and controls with a shorter observation time than 12 and 24 months respectively, and thereby limiting the healthy entrant effect and effect of referral bias, did not change the results significantly.

The clinical implications of this study are that we can safely continue to give reassuring messages to patients with NFAA. Even patients with discrete MACS (cortisol_DST_ 50-82 nmol/L) who are older or have a life expectancy of less than 10 years probably do not benefit from follow-up regarding their adrenal incidentalomas. As these groups constitute most patients with adrenal incidentalomas, implementing this knowledge in clinical care could significantly decrease unnecessary procedures and reduce patient worries.

The findings also emphasize that patients with cortisol_DST_ ≥83 nmol/L and younger patients with discrete MACS need follow-up and treatment of known risk factors for cardiovascular disease. Current guidelines on treatment of MACS are based on presently available studies, which offer low-quality evidence, leading to recommendations being based on extrapolated data and consensus statements (1, 30-32). Results from surgical studies are few and conflicting (33, 34). Perhaps pharmacological treatment with agents blocking the steroidogenesis/glucocorticoid effect could be another option. These drugs might also play a role in identifying patients who could benefit from surgery (35-38).

The overarching clinical questions for doctors seeing patients with MACS are whom and how to treat. This study further highlights the need for intervention studies, with hard endpoints, of patients with MACS.

In conclusion, NFAAs do not seem to pose a clinically relevant risk for increased 10-year mortality in patients with adrenal incidentalomas. This information allows physicians to reassure many patients with adrenal incidentalomas who do not need follow-up and focus the efforts of the health care system on the patients who are at increased risk, namely the MACS patients, especially younger patients, and those with a cortisol_DST_ level ≥83 nmol/L.

## Data Availability

Some or all datasets generated during and/or analyzed during the current study are not publicly available but are available from the corresponding author on reasonable request.

## Abbreviations

MACS: mild autonomous cortisol secretion
HR: hazard ratio
NFAA: nonfunctional adrenal adenomas
